# Assessment of a virtual reality temporal bone surgical simulator: a national face and content validity study

**DOI:** 10.1186/s40463-020-00411-y

**Published:** 2020-04-07

**Authors:** Evan C. Compton, Sumit K. Agrawal, Hanif M. Ladak, Sonny Chan, Monica Hoy, Steven C. Nakoneshny, Lauren Siegel, Joseph C. Dort, Justin T. Lui

**Affiliations:** 1 Section of Otolaryngology–Head and Neck Surgery, Department of Surgery, Cumming School of Medicine, University of Calgary, Calgary, Alberta Canada; 2 Department of Otolaryngology–Head and Neck Surgery, Western University, London, Ontario Canada; 3 Department of Electrical and Computer Engineering, Western University, London, Ontario Canada; 4 Department of Computer Sciences, University of Calgary, Calgary, Alberta Canada; 5 Ohlson Research Initiative, Arnie Charbonneau Cancer Institute, Cumming School of Medicine, University of Calgary, 3280 Hospital Dr. NW, Calgary, AB T2N 4Z6 Canada; 6 Department of Otolaryngology–Head and Neck Surgery, University of Toronto, Toronto, Ontario Canada; 7 Department of Medical Biophysics, Western University, London, Ontario Canada

**Keywords:** Face validity, Content validity, Virtual reality, Temporal bone, Dissection, Education, Patient-specific, Surgical simulation

## Abstract

**Background:**

Trainees in Otolaryngology–Head and Neck Surgery must gain proficiency in a variety of challenging temporal bone surgical techniques. Traditional teaching has relied on the use of cadavers; however, this method is resource-intensive and does not allow for repeated practice. Virtual reality surgical training is a growing field that is increasingly being adopted in Otolaryngology. CardinalSim is a virtual reality temporal bone surgical simulator that offers a high-quality, inexpensive adjunct to traditional teaching methods. The objective of this study was to establish the face and content validity of CardinalSim through a national study.

**Methods:**

Otolaryngologists and resident trainees from across Canada were recruited to evaluate CardinalSim. Ethics approval and informed consent was obtained. A face and content validity questionnaire with questions categorized into 13 domains was distributed to participants following simulator use. Descriptive statistics were used to describe questionnaire results, and either Chi-square or Fishers exact tests were used to compare responses between junior residents, senior residents, and practising surgeons.

**Results:**

Sixty-two participants from thirteen different Otolaryngology–Head and Neck Surgery programs were included in the study (32 practicing surgeons; 30 resident trainees). Face validity was achieved for 5 out of 7 domains, while content validity was achieved for 5 out of 6 domains. Significant differences between groups (*p*-value of < 0.05) were found for one face validity domain (realistic ergonomics, *p* = 0.002) and two content validity domains (teaching drilling technique, *p* = 0.011 and overall teaching utility, *p* = 0.006). The assessment scores, global rating scores, and overall attitudes towards CardinalSim, were universally positive. Open-ended questions identified limitations of the simulator.

**Conclusion:**

CardinalSim met acceptable criteria for face and content validity. This temporal bone virtual reality surgical simulation platform may enhance surgical training and be suitable for patient-specific surgical rehearsal for practicing Otolaryngologists.

## Background

Otolaryngology–Head and Neck Surgery (OHNS) is a surgical specialty that requires comprehensive training in many complex surgical skills [1]. One of the more challenging areas to gain proficiency in is temporal bone surgery [2]. Procedures involving the temporal bone require knowledge of complex micro-anatomy, use of an operating microscope, and avoidance of devastating complications [3, 4]. Unfortunately, the opportunities to perform otologic procedures in residency are decreasing and procedures are usually performed individually, making it difficult for trainees to learn in the operating room (OR) [4].

During OHNS residency training, there is a specific academic emphasis on becoming competent in cortical mastoidectomy through a combination of educational approaches [5]. Traditional teaching paradigms in OHNS combine didactic lectures with cadaveric dissection. Cadaveric dissections enable residents to practice surgical techniques in a controlled environment, and the use of a real drill and bone provides a high-fidelity experience for trainees.

Virtual reality (VR) simulation has many advantages over cadaveric dissection. Cadaveric specimens are associated with limited availability, single-use dissection, and absence of patient-specific pathology [6]. VR simulation has the ability to implement a range of virtual pathologies into limitless practice iterations [7]. A recent survey of Canadian training programs demonstrated that frequent, low-stakes practice is a perceived advantage of VR simulation surgery [8]. Moreover, resident trainee confidence has been shown to increase after virtual dissection, which is highly correlated with improved mastoidectomy performance [7]. VR temporal bone drilling has also been shown to be a more effective educational tool when introducing temporal bone surgery to novice users [9]. Training methods that combine the strengths of both cadaveric and VR dissection are feasible and potentially more effective than current cadaver-only approaches.

Before a simulation tool can be widely adopted for use in training programs, it must undergo validation [10]. The first two steps are: face validation, the degree to which the simulation resembles the real situation; and content validation, the degree to which the simulation deals with the subject matter of the real situation [10]. Previous studies assessing face and content validity of surgical simulators have presented mixed results possibly due to homogenous, single-centre sample of users [11–13]. To address this limitation, we present the first national face and content validity study of a VR temporal bone simulator.

Among surgical specialties, OHNS is a leader in simulation [7, 14, 15]. A recent systematic review by Musbashi et al. identified 64 different surgical simulators within OHNS. Thirty-two simulators were specific to ear and temporal bone surgery, with six being VR simulators [16]. Although the majority of otologic simulators focus on temporal bone surgery, other platforms exist including a VR myringotomy simulator that recently achieved face, content, and construct validity [17, 18]. Virtual reality simulators for temporal bone surgery are well documented and various groups have shown promising benefits for residency education [7, 11, 15, 19–22]. However, currently available commercial temporal bone simulators such as Voxel-Man® (Voxel-Man Group, Hamburg, Germany) are still prohibitively costly [23]. The present paper discusses CardinalSim, a temporal bone surgical simulator that may be a less costly alternative to other VR systems.

CardinalSim is an open-access VR temporal bone simulator software created at Stanford University [24]. Ongoing development of CardinalSim is part of a collaborative effort between Western University, University of Calgary, and Stanford University. CardinalSim utilizes patient-specific diagnostic imaging to generate a virtual specimen, upon which users can perform repeated surgical dissection. Resident trainees have previously reported increased confidence in cadaveric drilling after using CardinalSim (an average increase of 1.58 on 10-point confidence Likert scale, *p* < 0.01) and positive attitudes for using the simulator for patient-specific preoperative planning [22]. The high-fidelity haptic (force feedback) environment created using CardinalSim has been previously described and shown to enhance the training experience [25]. This national study explores the next critical steps in the development of CardinalSim: face and content validation.

## Methods

Participants from 13 different academic centers were recruited from the June 2019 Canadian Society of Otolaryngology–Head and Neck Surgery (CSOHNS) national meeting in Edmonton, Alberta. Participants were divided into two groups: practicing otolaryngologists and resident trainees. Residents were then subdivided into junior and senior levels according to postgraduate year (PGY). Junior residents were defined as being in PGY 1 and 2 and senior residents were in PGY 3, 4, and 5. Practicing otolaryngologists were subdivided based on their previous mastoidectomy surgical volumes, with greater than 10 mastoidectomies per year as the differentiating boundary between high-volume from low-volume surgeons. Ethics approval was obtained from the Western University Health Sciences Research Ethics Board (HSREB) and the University of Calgary Conjoint Health Research Ethics Board (CHREB).

Face and content validity testing were conducted at the national meeting using portable CardinalSim drilling stations. Temporal bone VR drilling stations were comprised of consumer-grade hardware systems using a Microsoft Windows 10 (Microsoft, Redmond, WA) operating system. Three-dimensional (3D) graphics were supported by NVIDIA® (Santa Clara, CA) graphics cards and NVIDIA® 3D Vision™ 2 glasses. Geomagic® (Morrisville, NC) Touch™ haptic devices served as the drill, which was paired with the 3Dconnexion SpaceMouse® Compact (Munich, DE) to simulate a first-person microscopic view in 3D space. Each temporal bone VR drilling station costs approximately 5000 Canadian dollars. The virtual temporal bone specimen used in the study was created using Digital Imaging and Communications in Medicine (DICOM) files from a human cadaveric specimen captured by a micro-computed tomography (CT) scanner. The detailed anatomy was segmented semi-automatically with 3D Slicer, an open-source software (www.slicer.org) [26]. The VR rendering was evaluated for accuracy by three experienced OHNS surgeons (SA, JCD, JTL).

Participants were instructed to perform a cortical mastoidectomy, including a posterior tympanotomy, during a 20-min drilling session. Participants underwent a 5-min introduction to the simulator and graduate students trained in using CardinalSim were present as surgical assistants to alleviate any technological issues. Therefore, no prior experience with CardinalSim was necessary for participation in the study.

After using CardinalSim, participants were asked to complete a face and content validity questionnaire using SurveyMonkey© (San Mateo, CA). The questionnaire was adapted from the surgical simulation literature [11, 12]. The questionnaire is provided in the supplementary materials. Items were rated using a 5-point Likert-type scale, with 1 representing “strongly disagree” and 5 representing “strongly agree.” In addition to face and content validity, the questionnaire included questions about the utility of the simulator to assess user performance as well as open-ended questions for general feedback about CardinalSim. Questions were categorized by domain, with 7 domains for face validity and 6 for content validity. Responses were reported as the median score for each domain, with a median of 4 or greater indicating validity for that domain. Free text feedback from the open-ended questions was independently summarized by three investigators (EC, JL, and SCN). Attending surgeons’ and residents’ results were subdivided based on years of experience.

The data were exported to Microsoft Excel (Microsoft, Redmond, WA) and analyzed using Stata IC version 16.0 (Stata Corp, College Station, TX). Chi-square or Fishers exact tests were used to compare responses between groups. A *p*-value of < 0.05 was considered significant.

## Results

### Demographics

Of the total 62 participants, 32 were attending OHNS surgeons, while 30 were resident trainees (21 junior residents, 9 senior residents). One OHNS fellow was included in the study and was grouped into the senior resident group. For the attending surgeons, 50% performed over 10 mastoidectomies per year, and 56% were in practice for 11 years or more. The characteristics of the participants, such as handedness, mastoidectomy experience, gender, and years of experience, are shown in Table 1. There were no significant differences between groups in gender distribution. The majority were right-handed (87% of resident trainees and 94% of attending surgeons). As expected, there were significant differences in the amount of mastoidectomies performed between resident trainees and attending surgeon groups (*p* < 0.0001). The participants included were from 13 different academic centers across Canada.

**Table 1:** The demographics of participating otolaryngologists and resident trainees

Participant Characteristics			
Number of Participants(%)			
**Characteristic**	**Resident Trainees*n*** **= 30**	**Attending Surgeons*n*** **= 32**	*p*-value
**Gender**			
Male	19 (63%)	25 (78%)	ns
Female	11 (37%)	7 (22%)	
**Handedness**			
Right	26 (87%)	30 (94%)	ns
Left	4 (13%)	2 (6%)	
**PGY Level**			
1	17 (57%)	–	–
2	4 (13%)	–	
3	6 (20%)	–	
4	1 (3%)	–	
5	1 (3%)	–	
Fellow	1 (3%)	–	
**Practice Years**			–
0–2	–	5 (16%)	
3–5	–	4 (13%)	
6–10	–	5 (16%)	
11+	–	18 (56%)	
**Mastoidectomies Performed**	**(total)**	**(per year)**	**< 0.0001**
0–2	21 (70%)	10 (31%)	
3–5	2 (7%)	4 (13%)	
6–10	4 (13%)	2 (6%)	
11–20	1 (3%)	16 (50%)	
21+	1 (3%)	0 (0%)	
Unknown	1 (3%)	0 (0%)	

### Face validity

**Fig. 1:**
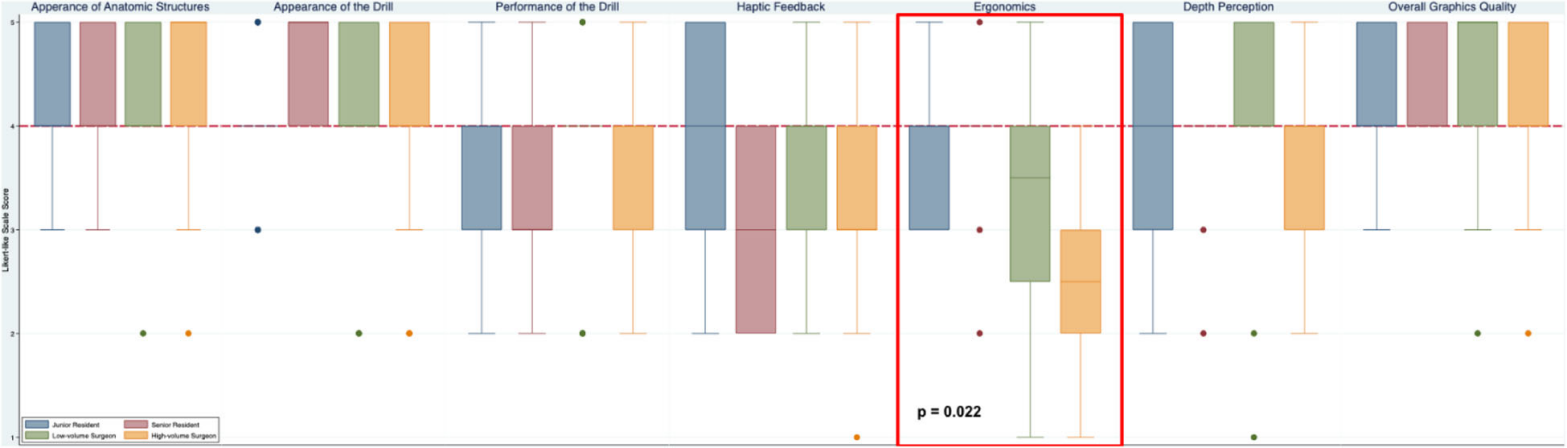
Face validity (realism) of CardinalSim assessed by otolaryngologists and resident trainees

The 7 domains of face validity (realism) included: the appearance of temporal bone anatomy and drill; performance of the drill; haptic feedback; ergonomics; depth perception; and overall graphics quality (Table 2). CardinalSim was considered realistic in 5 out of the 7 face validity domains (defined as a median of 4 or greater). High-volume surgeons strongly agreed (median of 5) that the appearance of the anatomical structures and drill were realistic. In Fig. 1, a panel of pictures is included to illustrate the realistic detail of temporal bone anatomy achieved using CardinalSim.

**Table 2:** The face validity scores by otolaryngologists and resident trainees

Face Validity					
Domains	Junior Trainees (PGY 1–2)	Senior Trainees (PGY 3+)	Low-volume Surgeons (0–10 Mastoids/year)	High-volume Surgeons (> 10 Mastoids/year)	*p*-value*
**Realistic Appearance of Anatomy**	A	A	A	SA	ns
**Realistic Appearance of Drill**	A	SA	A	SA	ns
**Realistic Performance of Drill**	A	A	A	A	ns
**Realistic Haptic Feedback**	N	A	A	N	ns
**Realistic Ergonomics**	N/A	A	A	D	**0.022**
**Realistic Depth Perception**	A	A	A	A	ns
**Realistic Overall Graphics Quality**	A	A	A/SA	A	ns

SA = Strongly Agree, A = Agree, N=Neutral, D = Disagree, ns = not significant

* chi-sqaure test, *p* ≤ 0.05 considered significant

**Fig. 2:**
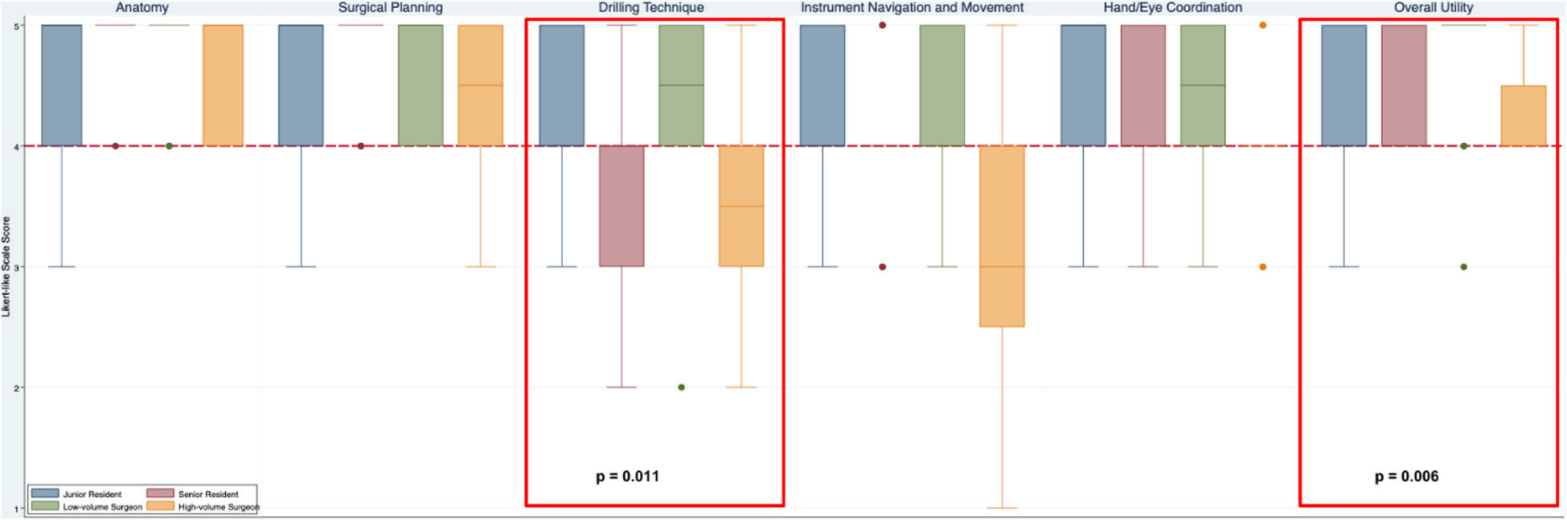
Content validity of CardinalSim assessed by otolaryngologists and resident trainees

The results for ergonomics and haptics were mixed. High-volume surgeons reported the lowest score for face validity in the ergonomics domain (median of 2.5), and there was significant disagreement with the other participant groups (*p* = 0.022). Senior residents and high-volume attending surgeons were neutral (median = 3) about CardinalSim haptics whereas junior residents and low-volume attending surgeons were more positive, reporting a median of 4 for haptics. All groups agreed that depth perception was realistic (median of 4). Fig. 2 illustrates the distribution of responses and median scores for each face validity domain for CardinalSim. A red dashed line indicates the cut-off for validity in each domain (median score of 4 or greater).

### Content validity

**Fig. 3:**
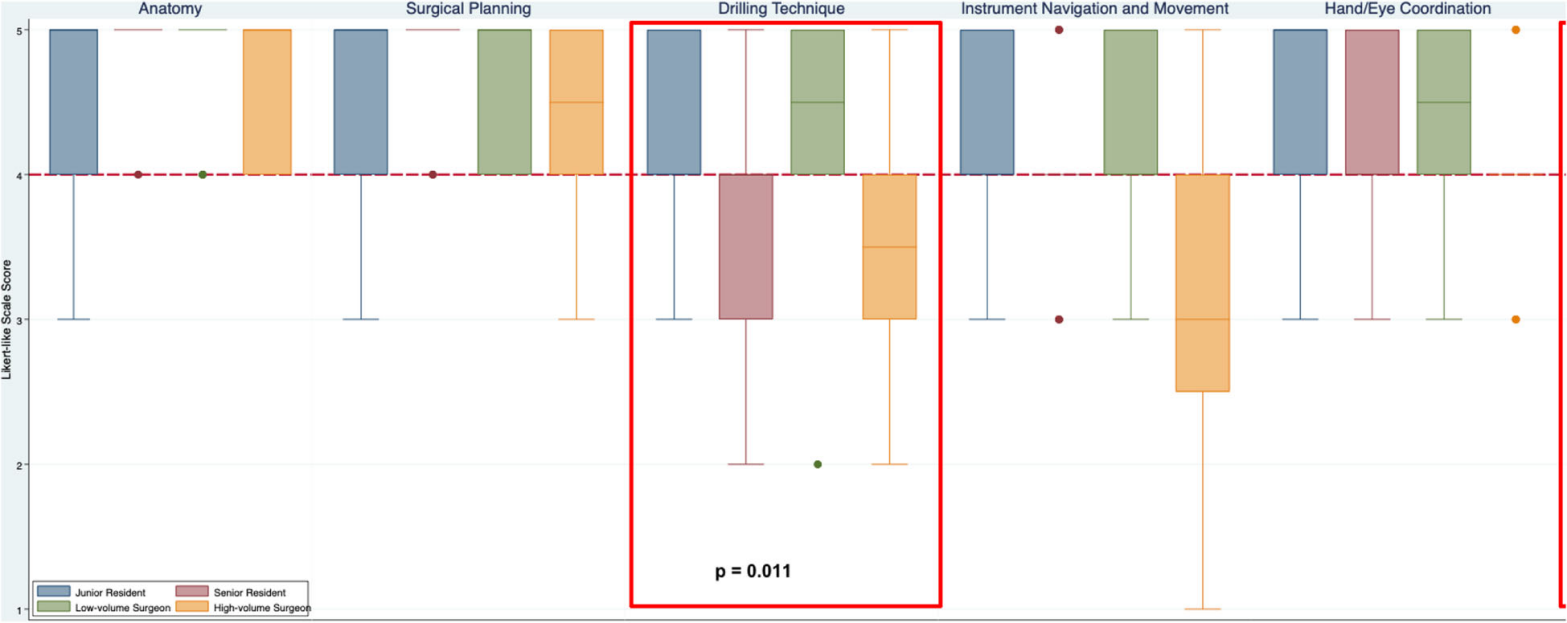
Content validity of CardinalSim assessed by otolaryngologists and resident trainees

The 6 domains of content validity (usefulness for teaching) included: teaching anatomy; surgical planning; drilling technique; instrument navigation; hand/eye coordination; and overall utility (Table 3). CardinalSim was considered useful by all residents and low-volume surgeons in 5 out of the 6 content validity domains (median of 4 or greater). However, results were mixed in the high-volume surgeon group. High-volume surgeons felt that CardinalSim was slightly less effective for teaching drilling technique and instrument navigation. For teaching anatomy and surgical planning, a median of 5 was reported by all groups. For teaching drilling technique, there was a significant difference between groups (*p* = 0.011). For instrument navigation and movement, high-volume attending surgeons reported a median of 3. The overall teaching quality was considered valid across all groups (median of 4 or greater) and the difference between groups was significant (*p* = 0.006). Fig. 3 is a box plot illustrating the distribution and median scores of each content validity domain CardinalSim. A red dashed line indicates the cut-off for validity in each domain (median score of 4 or greater).

**Table 3:** The content validity scores by otolaryngologists and resident trainees

Content Validity					
Domains	Junior Trainees (PGY 1–2)	Senior Trainees (PGY 3+)	Low-volume Surgeons (0–10 Mastoids/year)	High-volume Surgeons (> 10 Mastoids/year)	*p*-value*
**Useful for Teaching Anatomy**	SA	SA	SA	SA	ns
**Useful for Teaching Surgical Planning**	SA	SA	SA	SA	ns
**Useful for Teaching Drilling Technique**	SA	A	SA	A	**0.011**
**Useful for Teaching Instrument Navigation & Movement**	A	A	A	N	ns
**Useful for Teaching Hand/Eye Coordination**	SA	A	SA	A	ns
**Overall Teaching Utility**	SA	A	SA	A	**0.06**

SA = Strongly Agree, A = Agree, N=Neutral, ns = not significant

* chi-sqaure test, *p* ≤ 0.05 considered significant

### Global rating and assessment utility

Results of the global rating, defined as essential aspects about the use of CardinalSim, are summarized in Table 4. Questions pertained to CardinalSim’s applicability to residency training, ease of use, and potential skills transference to the operating room. All of the global rating and utility assessment questions reported a median of 4 or greater. There was significant disagreement between groups about whether skills acquired using CardinalSim are transferable to the OR (*p* = 0.035). Results of the assessment utility of CardinalSim are presented in Table 5 and include CardinalSim’s usefulness for assessment of drilling technique, instrument navigation, hand/eye coordination, and overall surgical skill. There was general agreement among all groups that CardinalSim was a useful assessment tool.

**Table 4:** The global rating scores by otolaryngologists and resident trainees

Global Rating					
Domains	Junior Trainees (PGY 1–2)	Senior Trainees (PGY 3+)	Low-volume Surgeons (0–10 Mastoids/year)	High-volume Surgeons (> 10 Mastoids/year)	*p*-value*
**CardinalSim Should be Included in Training**	SA	SA	SA	SA	ns
**Would Recommend CardinalSim to a Colleague**	SA	SA	SA	SA	ns
**CardinalSim is User-Friendly**	SA	SA	SA	A	ns
**CardinalSim Skills are OR Transferable**	SA	A/SA	SA	A	**0.035**

SA = Strongly Agree, A = Agree, ns = not significant

* chi-sqaure test, *p* ≤ 0.05 considered significant

**Table 5:** The utility of assessment rating by otolaryngologists and resident trainees

Assessment					
Domains	Junior Trainees (PGY 1–2)	Senior Trainees (PGY 3+)	Low-volume Surgeons (0–10 Mastoids/year)	High-volume Surgeons (> 10 Mastoids/year)	*p*-value*
**Useful for Assessment of Drilling Technique**	A	A	A	A	ns
**Useful for Assessment of Instrument Navigation & Movement**	A	A	A	A	ns
**Useful for Assessment of Hand/Eye Coordination**	A/SA	A	A	A	ns
**Useful for Assessment of Overal Ability**	A	A	A/SA	A	ns

SA = Strongly Agree, A = Agree, ns = not significant

* chi-sqaure test, *p* ≤ 0.05 considered significant

### Open-ended feedback

Results from the open-ended questions were separated by participant group and are summarized in Table 6. Participants frequently mentioned that CardinalSim would be useful as a patient-specific preoperative planning and resident education tool. The haptics and ergonomics of the simulator were listed frequently as limitations of CardinalSim, which aligns with the face validity results. Otolaryngologists and resident trainees reported decreased OR time and reduced surgical complications as potential benefits of CardinalSim. Respondents also stated that the best time for simulator rehearsal of surgical cases was 24–48 h before a planned procedure and that a clinic setting was best for such rehearsal. Additionally, residents reported increased confidence in their temporal bone drilling after using CardinalSim and suggested there might be a role for CardinalSim during patient education and the informed consent process. Finally, otolaryngologists reported that an advantage of utilizing CardinalSim would be a substantial reduction in the cost of maintaining a cadaveric temporal bone laboratory.

**Table 6:** Open-ended feedback from otolaryngologists and resident trainees

Open-ended question summary		
**Questions**	**Resident Trainees**	**Attending Surgeons**
**Advantages of CardinalSim**	Learning anatomy, teaching anatomy, patient-specific practice	Reduce cadaveric laboratory costs, teaching residents (emphasis on junior), patient-specific drilling
**Limitations of CardinalSim**	Ergonomics, haptics, lack of soft tissue structures (blood, fascia, skin, muscles)	Haptics, no simulation of blood, ergonomics
**Role in patient consent and education**	Generally agree with potential role	Mixed response
**Timing / location of pre-operative drilling**	24–48 h before OR / Clinic	24–48 h before OR / Clinic
**Potential benefits of CardinalSim**	Increased confidence, reduced OR times, reduced complications	Reduced OR time, reduced complicatons

## Discussion

In this national study, we quantified face and content validity for CardinalSim, an open-access VR temporal bone surgery simulator. Otolaryngology surgeons and resident trainees from across Canada were recruited to participate in testing and assessment of the technology. Face and content validity for CardinalSim were achieved in most domains. Additionally, the global rating of CardinalSim by respondents was strongly positive across all domains. Specifically, all respondents stated that they strongly agreed that CardinalSim should be used in residency training to enhance surgical education and would recommend CardinalSim to a colleague. Furthermore, the perception of skills transference to the OR was unanimously agreed upon by respondents.

The ergonomics and haptic feedback of CardinalSim were two domains that lacked acceptable validity scores. Specifically, high-volume attending surgeons reported the ergonomics were not realistic. Common ergonomic complaints were arm fatigue and one-handed simulation, which will be addressed in future iterations of the software with the addition of a foot pedal and armrests. Achieving validity for ergonomics and haptic feedback has also been challenging for other temporal bone simulator research groups [11, 12]. Importantly, highly realistic haptics and ergonomics might not be critical for a high-quality training tool. Arora et al. suggested that realism is of lesser importance for novice trainees since content validity and positive global attitudes for their temporal bone simulator was independent of face validity [12]. Similarly, present findings revealed that despite partially realistic ergonomics and haptics, respondents would still value CardinalSim for preoperative planning, teaching, and resident assessment. Other research groups have argued for using 3D printed temporal bones over VR technology because of the presence of tactile feedback [13, 27]. For instance, Hochman et al. are developing a mixed reality dissection simulator citing enhanced haptic realism using 3D printed temporal bone specimens [27]. Simulators that involve 3D printed technology do have an inherent advantage for haptic realism compared to VR platforms; however, these technologies are expensive and lack repeated dissection and real-time automated evaluation.

In addition to surgical training, CardinalSim may prove ideal for assessment of OHNS residents’ temporal bone knowledge and surgical ability. Currently, there is no standardized assessment of residents in Canada for cortical mastoidectomy, despite it being a crucial educational competency. Moreover, residency program directors and residents have acknowledged that a revamp of current temporal bone surgical teaching is desirable [8]. The present participants reported that CardinalSim should be utilized in residency programs for teaching and evaluation of drilling techniques, instrument navigation, and hand-eye coordination. Therefore, CardinalSim is well-positioned for implementation into residency programs to enhance temporal bone surgery training and evaluation. Implementing CardinalSim within the current demands of residency training might be challenging due to existing time constraints; however, the results of the present study indicate that it would be a welcomed tool by residents. Furthermore, the ability for CardinalSim to standardize trainee evaluation has the potential to improve patient care and safety through increased preparedness and confidence of residents.

Another useful application of CardinalSim is case-specific surgical rehearsal for preoperative planning. This interactive technology is complimentary to the static nature of traditional preoperative diagnostic imaging review. Although reviewing CT scans is the gold-standard, the reliability of predicting operative findings in patients with temporal bone disease is imperfect [28]. Badran et al. found that operative findings are congruent with interpretations of CT temporal bone scans less than 80% of the time [28]. Specifically, facial canal dehiscence reporting is found to be only 60% accurate [28]. In contrast, Chan et al. showed a high level of accuracy for CardinalSim virtual reality renderings in patient-specific CT scans when compared to intraoperative findings [25].Bartling et al. described increased detail of the facial nerve using a fusion of CT and magnetic resonance imaging (MRI), but this modality lacks the added advantage of expanded exploration of a patient’s anatomy through repeated virtual dissection [29]. CardinalSim has the potential to complement current pre-operative review of CT imaging by providing an interactive three-dimensional interface.

Compared to other VR temporal bone simulators, CardinalSim fared favourably in subjective realism [11, 12, 30]. Content validity was achieved in the Voxel-Man® simulator, but face validity was not achieved due to considerable hesitation regarding the realism of the software [12]. Varoquier et al. demonstrated similar results for Voxel-Man®; however, they also showed the simulator could distinguish between novice and experienced users based on surgical ability using an automatic assessment tool [11]. A study is currently underway for CardinalSim to undergo testing of an integrated automatic assessment tool and preliminary feedback is positive. Wiet et al. explored face and content validity of the Ohio State VR temporal bone simulator but did not share specific validity survey results [31]. Our CardinalSim face and validity results prove the technology is comparable with other industry leaders and that CardinalSim has a competitive edge in realism.

This study also demonstrated some limitations. For the resident cohort, a disproportionate number of residents were junior trainees, who may lack adequate experience to rate the realism of a temporal bone simulator. However, there were not significant differences between cohorts for most domains. Furthermore, the evaluation of CardinalSim by low and high-volume surgeons demonstrate face and content validation for the majority of domains (Tables 2 and 3). Common complaints about CardinalSim included suboptimal haptic feedback and ergonomics which agreed with face validity results. Participants cited the lack of simulated blood and soft tissues as disadvantages, which are common limitations of other simulators in the literature [12]. CardinalSim is designed to create a realistic representation of complex anatomy for surgical training without distractions, such as simulated blood. Additionally, the face validity results confirm that realism is not negatively impacted by a lack of blood or debris. Therefore, while this type of aesthetic detail might be appealing, it is not necessary for an impactful educational experience with VR temporal bone surgery.

## Conclusion

CardinalSim met acceptable standards for face and content validity. For the first time in VR temporal bone drilling research and development, surgical simulation validity results were gathered on a national scale. Considerable interest within the OHNS community exists for adaptation of CardinalSim to postgraduate education and clinical practice. An opportunity exists to improve residency training in temporal bone drilling by creating a hybrid model of combined VR and cadaveric experience. By demonstrating face and content validity, a proper skills transference study can be conducted to see if CardinalSim is an effective tool in residency training and surgical rehearsal.

## Data Availability

The datasets during and/or analysed during the current study available from the corresponding author on reasonable request.

## References

[1] Wagner N, Fahim C, Dunn K, Reid D, Sonnadara RR (2017). Otolaryngology residency education: a scoping review on the shift towards competency-based medical education. Clin Otolaryngol.

[2] Andersen SAW (2016). Virtual reality simulation training of mastoidectomy - studies on novice performance. Dan Med J.

[3] Wiet GJ, Sørensen MS, Andersen SAW (2017). Otologic skills training. Otolaryngol Clin North Am.

[4] Malik MU, Varela D, Park E (2013). Determinants of resident competence in mastoidectomy: role of interest and deliberate practice. Laryngoscope..

[5] Kashikar TS, Kerwin TF, Moberly AC, Wiet GJ. A review of simulation applications in temporal bone surgery. Laryngoscope Investig Otolaryngol. 2019:1–5. 10.1002/lio2.277. 10.1002/lio2.277 PMC6703115 31453352

[6] Naik SM, Naik MS, Bains NK (2014). Cadaveric temporal bone dissection: is it obsolete today?. Int Arch Otorhinolaryngol.

[7] Lui JT, Hoy MY (2017). Evaluating the effect of virtual reality temporal bone simulation on Mastoidectomy performance: a meta-analysis. Otolaryngol Head Neck Surg.

[8] Lui JT, Compton ED, Ryu WHA, Hoy MY (2018). Assessing the role of virtual reality training in Canadian Otolaryngology – Head & Neck Residency Programs : a national survey of program directors and residents.

[9] Andersen SAW, Mikkelsen PT, Konge L, Cayé-Thomasen P, Sørensen MS (2016). Cognitive load in Mastoidectomy skills training: virtual reality simulation and traditional dissection compared. J Surg Educ.

[10] Carter FJ, Schijven MP, Aggarwal R (2005). Consensus guidelines for validation of virtual reality surgical simulators. Surg Endosc Other Interv Tech.

[11] Varoquier M, Hoffmann CP, Perrenot C, Tran N, Parietti-Winkler C (2017). Construct, face, and content validation on voxel-man® simulator for Otologic surgical training. Int J Otolaryngol.

[12] Arora A, Khemani S, Tolley N (2012). Face and content validation of a virtual reality temporal bone simulator. Otolaryngol Head Neck Surg.

[13] Mick PT, Arnoldner C, Mainprize JG, Symons SP, Chen JM (2013). Face validity study of an artificial temporal bone for simulation surgery. Otol Neurotol.

[14] Deutsch ES, Wiet GJ, Seidman M, Hussey HM, Malekzadeh S, Fried MP (2015). Simulation activity in otolaryngology residencies. Otolaryngol Head Neck Surg.

[15] Arora A, Swords C, Khemani S (2014). Virtual reality case-specific rehearsal in temporal bone surgery: a preliminary evaluation. Int J Surg.

[16] Musbahi O, Aydin A, Al Omran Y, Skilbeck CJ, Ahmed K (2017). Current status of simulation in otolaryngology: a systematic review. J Surg Educ.

[17] Huang C, Cheng H, Bureau Y, Ladak HM, Agrawal SK (2018). Automated metrics in a virtual-reality Myringotomy simulator: development and construct validity. Otol Neurotol.

[18] Huang C, Cheng H, Bureau Y, Agrawal SK, Ladak HM (2015). Face and content validity of a virtual-reality simulator for myringotomy with tube placement. J Otolaryngol Head Neck Surg.

[19] Zirkle M, Roberson DW, Leuwer R, Dubrowski A (2007). Using a virtual reality temporal bone simulator to assess otolaryngology trainees. Laryngoscope..

[20] Zhao YC, Kennedy G, Yukawa K, Pyman B, O’Leary S (2011). Improving temporal bone dissection using self-directed virtual reality simulation: results of a randomized blinded control trial. Otolaryngol Head Neck Surg.

[21] Francis HW, Malik MU, Diaz Voss Varela DA (2012). Technical skills improve after practice on virtual-reality temporal bone simulator. Laryngoscope..

[22] Locketz GD, Lui JT, Chan S (2017). Anatomy-specific virtual reality simulation in temporal bone dissection: perceived utility and impact on surgeon confidence. Otolaryngol - Head Neck Surg (United States).

[23] Roushdi I, Tennent D (2015). Current usage patterns of procedure-based assessments in the orthopaedic community. Bull R Coll Surg Engl.

[24] Chan S, Li P, Lee DH, Salisbury JK, Blevins NH (2011). A virtual surgical environment for rehearsal of tympanomastoidectomy. Stud Health Technol Inform.

[25] Chan S, Li P, Locketz G, Salisbury K, Blevins NH (2016). High-fidelity haptic and visual rendering for patient-specific simulation of temporal bone surgery. Comput Assist Surg.

[26] Fedorov A, Beichel R, Kalpathy-cramer J (2012). 3D slicer as an image computing platform for the quantitative imaging network. Magn Reson Imaging.

[27] Hochman JB r, Sepehri N, Rampersad V (2014). Mixed reality temporal bone surgical dissector: mechanical design. J Otolaryngol Head Neck Surg.

[28] Badran K, Ansari S, Al Sam R, Al Husami Y, Iyer A (2016). Interpreting pre-operative mastoid computed tomography images: comparison between operating surgeon, radiologist and operative findings. J Laryngol Otol.

[29] Bartling SH, Peldschus K, Rodt T (2005). Registration and fusion of CT and MRI of the temporal bone. J Comput Assist Tomogr.

[30] Surgery N (2002). Virtual temporal bone dissection: An interactive surgical simulator.

[31] Evans CH, Schenarts KD (2016). Evolving educational techniques in surgical training. Surg Clin North Am.

