# Correction to: Assessment of a virtual reality temporal bone surgical simulator: a national face and content validity study

**DOI:** 10.1186/s40463-020-00416-7

**Published:** 2020-04-22

**Authors:** Evan C. Compton, Sumit K. Agrawal, Hanif M. Ladak, Sonny Chan, Monica Hoy, Steven C. Nakoneshny, Lauren Siegel, Joseph C. Dort, Justin T. Lui

**Affiliations:** 1 Section of Otolaryngology–Head and Neck Surgery, Department of Surgery, Cumming School of Medicine, University of Calgary, Calgary, Alberta Canada; 2 Department of Otolaryngology–Head and Neck Surgery, Western University, London, Ontario Canada; 3 Department of Electrical and Computer Engineering, Western University, London, Ontario Canada; 4 Department of Computer Sciences, University of Calgary, Calgary, Alberta Canada; 5 Ohlson Research Initiative, Arnie Charbonneau Cancer Institute, Cumming School of Medicine, University of Calgary, 3280 Hospital Dr. NW, Calgary, AB T2N 4Z6 Canada; 6 Department of Otolaryngology–Head and Neck Surgery, University of Toronto, Toronto, Ontario Canada; 7 Department of Medical Biophysics, Western University, London, Ontario Canada

**Correction to: J Otolaryngol Head Neck Surg**

**https://doi.org/10.1186/s40463-020-00411-y**

Following publication of the original article [1], the authors identified incorrect ordering and incorrect files being used for Figs. 1, 2 and 3.

The correct Figs. 1, 2 and 3 have been included in this Correction, and the original article has been corrected.

**Fig. 1:**
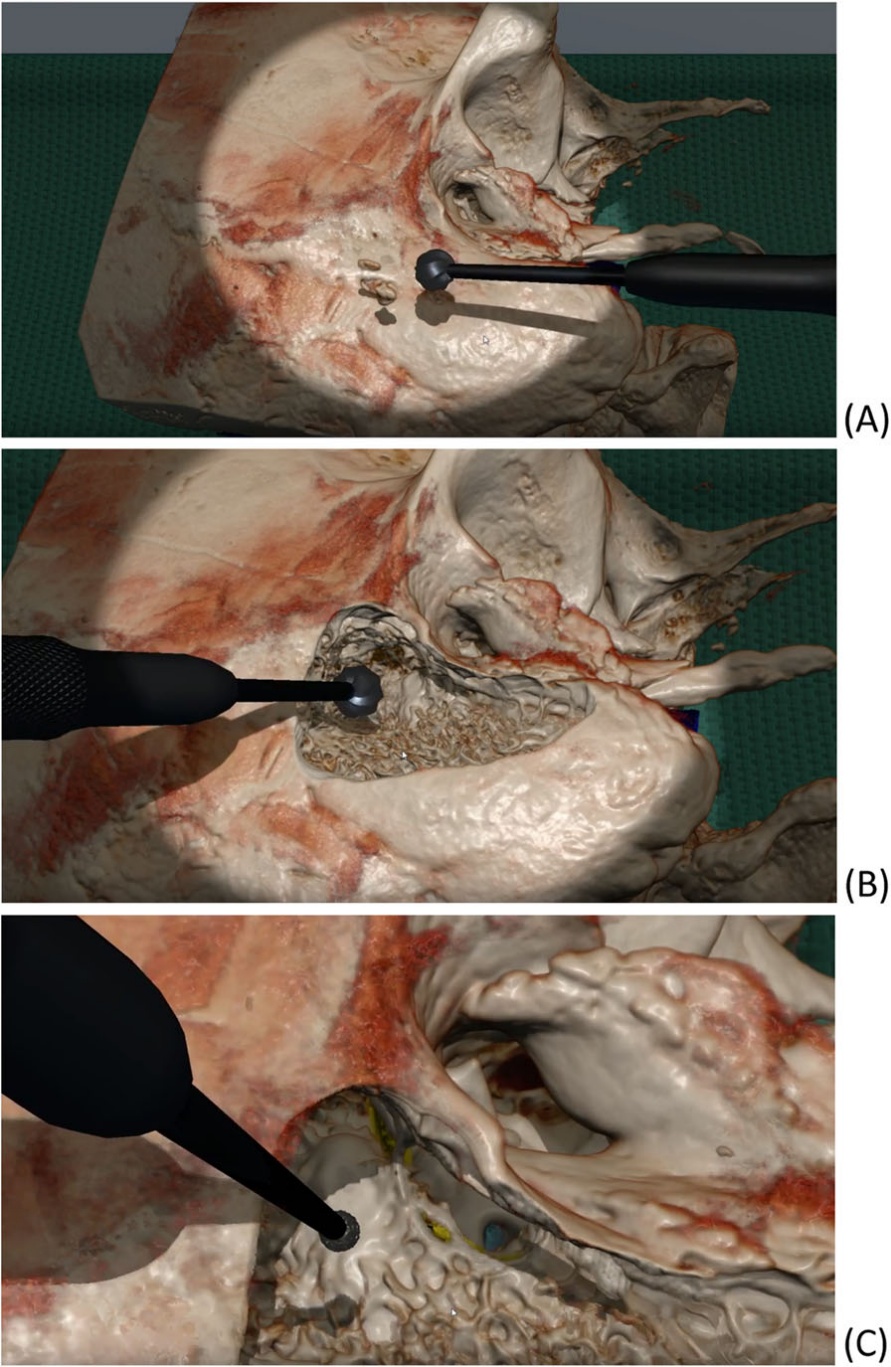
Screenshots of CardinalSim at varying stages of tympanotomy dissection. **a** Pre-dissection virtual reality segmented human cadaveric temporal bone. **b** Mid-dissection exposure of lateral semicircular canal completed with cutting burr drill. **c** Facial recess approach to round window completed with diamond burr drill, short process of incus and facial nerve (yellow) identified

**Fig. 2:**
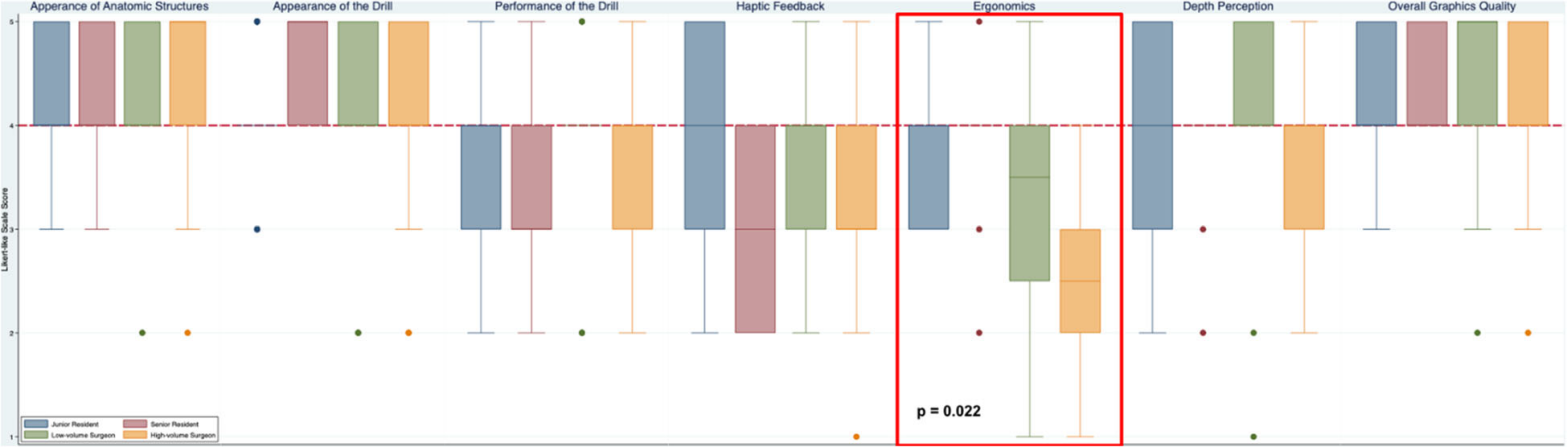
Face validity (realism) of CardinalSim assessed by otolaryngologists and resident trainees

**Fig. 3:**
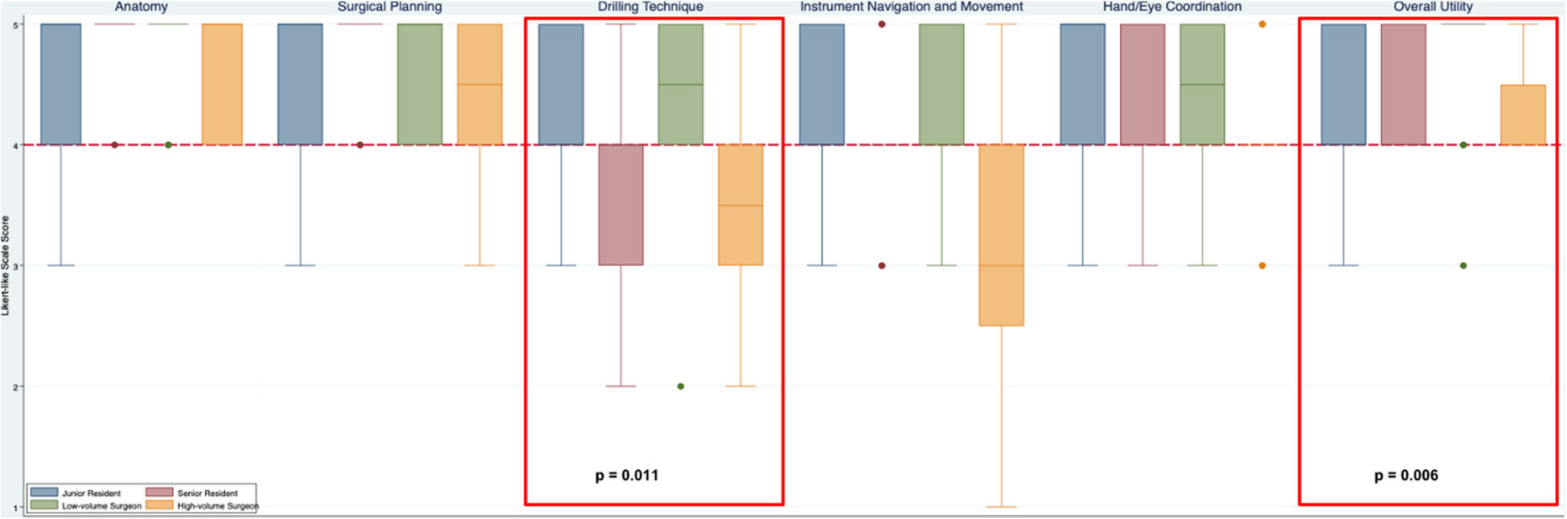
Content validity of CardinalSim assessed by otolaryngologists and resident trainees
